# Use of a Belatacept-based Immunosuppression for Kidney Transplantation From Donors After Circulatory Death: A Paired Kidney Analysis

**DOI:** 10.1097/TXD.0000000000001615

**Published:** 2024-04-11

**Authors:** Rita Eid, Anne Scemla, Magali Giral, Nadia Arzouk, Dominique Bertrand, Marie-Noëlle Peraldi, Laurent Mesnard, Helene Longuet, Mehdi Maanaoui, Geoffroy Desbuissons, Edouard Lefevre, Renaud Snanoudj

**Affiliations:** 1 Department of Nephrology and Transplantation, Bicêtre Hospital, Assistance Publique des Hôpitaux de Paris (AP-HP), Le Kremlin Bicêtre, France.; 2 Department of Nephrology and Transplantation, Necker University Hospital for Sick Children, AP-HP, Paris, France.; 3 Department of Nephrology and Transplantation, Nantes University Hospital Centre, Nantes, France.; 4 Department of Nephrology and Transplantation, Pitié Salpêtrière University Hospital, AP-HP, Paris, France.; 5 Department of Nephrology and Transplantation, Rouen University Hospital Centre, Rouen, France.; 6 Department of Nephrology and Transplantation, Saint-Louis Hospital, AP-HP, Paris, France.; 7 Department of Nephrology and Transplantation, Tenon Hospital, AP-HP, Paris, France.; 8 Department of Nephrology and Transplantation, Tours University Hospital Centre, Tours, France.; 9 Department of Nephrology and Transplantation, Lille University Hospital Centre, Lille, France.

## Abstract

**Background.:**

Efficacy and safety of belatacept have not been specifically reported for kidney transplantations from donors after circulatory death.

**Methods.:**

In this retrospective multicenter paired kidney study, we compared the outcome of kidney transplantations with a belatacept-based to a calcineurin inhibitor (CNI)-based immunosuppression. We included all kidney transplant recipients from donors after uncontrolled or controlled circulatory death performed in our center between February 2015 and October 2020 and treated with belatacept (n = 31). The control group included the recipients of the contralateral kidney that were treated with CNI in 8 other centers (tacrolimus n = 29, cyclosporine n = 2).

**Results.:**

There was no difference in the rate of delayed graft function. A higher incidence of biopsy-proven rejections was noted in the belatacept group (24 versus 6 episodes). Estimated glomerular filtration rate (eGFR) was significantly higher in the belatacept group at 3-, 12-, and 36-mo posttransplant, but the slope of eGFR was similar in the 2 groups. During a mean follow-up of 4.1 y, 12 patients discontinued belatacept and 2 patients were switched from CNI to belatacept. For patients who remained on belatacept, eGFR mean value and slope were significantly higher during the whole follow-up. At 5 y, eGFR was 80.7 ± 18.5 with belatacept versus 56.3 ± 22.0 mL/min/1.73 m^2^ with CNI (*P* = 0.003). No significant difference in graft and patient survival was observed.

**Conclusions.:**

The use of belatacept for kidney transplants from either uncontrolled or controlled donors after circulatory death resulted in a better medium-term renal function for patients remaining on belatacept despite similar rates of delayed graft function and higher rates of cellular rejection.

Transplantation of kidneys from donors after uncontrolled circulatory death (uDCD—Maastricht 2 category) and controlled circulatory death (cDCD—Maastricht 3 category) has emerged in the last 20 y to increase the pool of kidney donors. In France, procurement of kidneys has begun in 2006 for uDCD and in 2015 for cDCD. They accounted in 2021 for 0.3% and 11.9% of kidney transplantations, respectively.^1,2^ Normothermic renal perfusion was used as an in situ perfusion modality for this type of donors in France. Kidneys were immediately perfused on hypothermic machine perfusion after procurement.

Belatacept, an inhibitor of the CD28^−^CD80/86 costimulatory pathway, has been approved as an immunosuppressive drug in kidney transplantation, an alternative to calcineurin inhibitors (CNIs) in combination with steroids and mycophenolic acid.^3,4^ This costimulatory blockade strategy avoids side effects associated with CNI use (cardiovascular, metabolic, and renal toxicities) with a long-term improvement of overall graft survival, despite a higher rate of acute cellular rejection.^5^

Most of the studies showed a renal benefit of belatacept over cyclosporine in transplants from living and standard criteria donors but also for extended criteria donor kidneys.^6,7^ To date, there is no published study evaluating the efficacy and safety of belatacept compared with CNI for transplantation from DCD, either cDCD or uDCD.

In our transplant center, we use belatacept-based immunosuppression since 2015 for all Epstein-Barr virus-seropositive kidney transplant recipients from cDCD and uDCD, aiming to avoid short-term and long-term nephrotoxic effects of CNI that could be detrimental for this kind of transplantations. Contrary to the usual protocol, we use thymoglobulin induction rather than interleukin 2-receptor blockers, to reduce the risk of acute rejection.

We conducted a retrospective paired study that aimed to evaluate the safety and the efficacy of a belatacept compared with a CNI-based (mostly tacrolimus) immunosuppression. The paired kidney design was chosen to minimize the donor-effect and to focus on the treatment-effect.

## MATERIALS AND METHODS

### Inclusion of Patients and Immunosuppressive Protocol

We included all Epstein-Barr virus-seropositive recipients of a first kidney transplant from DCD who received, in our center, a belatacept-based immunosuppression between February 2015 and October 2020 (“belatacept group”), and whose contralateral kidney was transplanted with a CNI-based immunosuppression. The “CNI group” included the recipients of the contralateral kidney who received a CNI-based immunosuppression in 8 other transplantation centers. All patients should have achieved at least 1 y of follow-up.

The immunosuppressive protocol in the belatacept group was the “less-intensive protocol” used in the BENEFIT (Belatacept Evaluation of Nephroprotection and Efficacy as First‐line Immunosuppression Trial) study,^4^ with the exception of the induction that consisted of thymoglobulin (rabbit antithymocyte globulins), 1.25 mg/kg/d for 5 consecutive days. The initial immunosuppressive protocol in the CNI group was the local standard of care, combining thymoglobulin or basiliximab, tacrolimus or cyclosporine, mycophenolate mofetil and steroids.

Patients with preformed DSA (mean fluorescence intensity [MFI] between 1000 and 3000) were treated with intravenous immunoglobulin in addition to thymoglobulin in both groups.

### Clinical Data and Evaluation of Renal Function

Data regarding recipients and donors was retrospectively collected through chart review. For the assessment of renal function, we collected serum creatinine and estimated GFR (eGFR) through the 4-variables MDRD formula. Delayed graft function (DGF) was defined as the need for dialysis during the first week after transplantation. The day of graft function recovery was defined as the first day of sustained spontaneous creatinine reduction during at least 2 consecutive days. “The delay of graft function recovery” was calculated from the time span between the day of transplantation and the day of graft function recovery. All cellular and antibody-mediated rejection (AMR) episodes were biopsy-proven and classified according to the updated Banff classification.

Patients were screened monthly for BK-virus and cytomegalovirus (CMV) viremia during the first year. In the case of positive BK viremia, a kidney biopsy was performed to ascertain the diagnosis of BK-virus nephropathy. Screening of donor-specific antibodies (DSAs) was performed at 3 and 12 mo posttransplant, and annually thereafter. DSAs were considered de novo if they were absent before transplantation and if MFI was at least once ≥1000 with the Luminex single antigen technique.

### Outcomes

Patients were followed from the date of transplantation to May 1, 2022. Our main objective was to compare the evolution of renal function in the 2 groups with a paired analysis based on the shared donor, in the whole groups and stratified on the type of DCD (cDCD and uDCD). Evolution of renal function was assessed through the rate of DGF, the day of graft function recovery, serum creatinine (at days 7, 14, 28, months 3, 6, 12, and annually thereafter) and eGFR (at months 3, 6, 12, and annually thereafter). Secondary outcomes included biopsy-proven rejection episodes, graft and patient survivals, the 1-y incidence of positive BK viremia and CMV viremia, BK-virus nephropathy, and CMV disease.

### Statistical Analysis

Donor and recipient characteristics were described by means and SDs or frequencies, as appropriate. Comparisons of recipient characteristics between the 2 groups were performed with the Student’s *t*-test (or Wilcoxon tests for nonparametric variables) or the chi-square test. For paired comparisons of outcomes based on the donor, paired t-test were used only when the difference was normally distributed (verification with Shapiro–Wilk test). For categorical variables, the McNemar test was performed. Survival was estimated by the Kaplan–Meier method. The test for equality of survival curves was performed with the log-rank test (*P* value <0.05).

To study the impact of treatment in the evolution of renal function, we run a mixed-linear model in which treatment and time were considered as fixed effects, and individuals as random effects in terms of intercept and variation with time (or slope).

Final statistical significance was identified by a *P* value <0.05. Statistical analyses were performed using R software.

## RESULTS

### Baseline Characteristics of Recipients and Donors

Sixty-two patients were enrolled in our study (Table 1): 31 patients were treated with belatacept (belatacept group) and 31 patients with CNI (CNI group, tacrolimus in 29 and cyclosporine in 2 patients). There was no difference in terms of sex, age, nephropathy, medical history, and immunization. Preexisting DSA with MFI ≥1000 were present in 9.6% of patients in the belatacept group versus 12.9% in the CNI group but were directed against different HLA loci: B, Cw and DQ in the belatacept group, Cw and DP in the CNI group. MFI was <3000 for all preformed DSA.

**TABLE 1.** Characteristics of kidney transplant recipients in the belatacept and CNI groups

Recipients	Overall	CNI	Belatacept	*P*
N	62	31	31	
Sex, male, n (%)	47 (75.8)	25 (80.6)	22 (71.0)	0.553
Age, y, mean (SD)	50.6 (12.6)	49.9 (12.2)	51.3 (13.2)	0.675
Nephropathy, n (%)				0.127
Diabetic	9 (14.5)	4 (12.9)	5 (16.1)	
Glomerular	16 (25.8)	11 (35.5)	5 (16.1)	
Nephroangiosclerosis	11 (17.7)	5 (16.1)	6 (19.4)	
Undetermined	8 (12.9)	1 (3.2)	7 (22.6)	
Other	18 (29.0)	10 (32.3)	8 (25.8)	
Modality of dialysis (%)				0.355
PD	2 (3.2)	2 (6.5)	0 (0.0)	
HD	54 (87.1)	26 (83.9)	28 (90.3)	
No dialysis	6 (9.7)	3 (9.7)	3 (9.7)	
Mean duration on HD before KT, mo	40.6	39.9	41.3	0.870
cPRA, mean (SD)	13.0 (20.8)	11.3 (17.0)	14.7 (24.3)	0.519
Preformed DSA (%)	7 (11.3)	4 (12.9)	3 (9.6)	0.694
Diabetes (%)	16 (25.8)	10 (32.3)	6 (19.4)	0.384
Hypertension (%)	51 (82.3)	23 (74.2)	28 (90.3)	0.184
Dyslipidemia (%)	14 (23.0)	4 (13.3)	10 (32.3)	0.146
CMV-seropositive (%)	41 (66.1)	17 (54.8)	24 (77.4)	0.107
EBV-seropositive (%)	61 (98.4)	30 (96.8)	31 (100.0)	1.000

CNIs, calcineurin inhibitors; cPRA, calculated panel-reactive antibody; DSA, donor-specific antibody; EBV, Epstein-Barr virus; HD, hemodialysis; KT, kidney transplantation; PD, peritoneal dialysis.

Donors were procured after uncontrolled circulatory death (uDCD) in 48% of cases or after controlled circulatory death (cDCD) in 52% of cases (Table 2). Cold ischemia time was similar in the 2 groups: 780 (belatacept) versus 725 min (CNI), *P* = 0.13.

**TABLE 2.** Characteristics of kidney transplant donors

Donors	
N	31
Category of donors	
uDCD (%)	15 (48)
cDCD (%)	16 (52)
Hypertension	3 (9.6)
Diabetes	1 (3.2)
Sex	
Male (%)	23 (74)
Female (%)	8 (26)
Age, y, mean (SD) [min–max]	43 (11.4) [19–65]
Body mass index, mean (SD)	25 (5.4)
Cerebrovascular accident (%)	7 (22.6)
Cardiac arrest in cDCD (%)	4 (12.9)
Peak serum creatinine (µmol/L), mean (SD)	102 (36)
Last serum creatinine((µmol/L), mean (SD)	93 (40)
Causes of death	
Trauma (%)	5 (16.1)
Cardiac (%)	7 (22.6)
Cerebrovascular (%)	6 (19.4)
Other (%)	13 (41.9)

cDCD, donor after controlled circulatory death; CNIs, calcineurin inhibitors; uDCD, donor after uncontrolled circulatory death.

In the belatacept group, 12 patients (38.7%) were switched to another immunosuppressive drug: tacrolimus (n = 10 including 7 during the first year), everolimus (n = 1), and discontinuation for steroid monotherapy (n = 1). Causes of switch and medical events in the belatacept group are shown in Figure S1 (**SDC**, http://links.lww.com/TXD/A638). In the CNI group, 2 patients (6.4%) were switched from tacrolimus to belatacept for graft dysfunction at 8 and 26 mo posttransplantation.

### Evolution of Graft Function

DGF was observed in 22.6% and 29% of patients in the belatacept and CNI groups, respectively (*P* = 0.72). The incidence was also similar whatever the category of donors, uDCD or cDCD (Table 3). Notably, only 1 DGF (CNI) was observed after transplant from cDCD. The delay of graft function recovery was also similar in the 2 groups, whatever the category of donors (Table 3).

**TABLE 3.** Frequency of delayed graft function and delay of graft function recovery in kidney transplant recipients from donors after uncontrolled and controlled circulatory death

	Belatacept	CNI	*P* ^*a*^
DGF for the 2 groups (%)	7/31 (22.6)	9/31 (29.0)	0.72
DGF in uDCD (%)	7/15 (46.7)	8/15 (53.3)	1
DGF in cDCD (%)	0 (0)	1/16 (6.7)	n.c.
Delay of function recovery for the 2 groups, d, mean (SD)	7.8 (8.5)	6.4 (7.6)	0.31
Delay of recovery in uDCD, mean (SD)	14.9 (7.1)	11.5 (8.1)	0.24
Delay of recovery in cDCD, mean (SD)	1.2 (0.8)	1.6 (1.3)	0.28

^*a*^MacNemar and paired Student’s *t*-tests.

cDCD, donor after controlled circulatory death; CNIs, calcineurin inhibitors; DGF, delayed graft function; n.c., not calculable; uDCD, donor after uncontrolled circulatory death.

**FIGURE 1.**
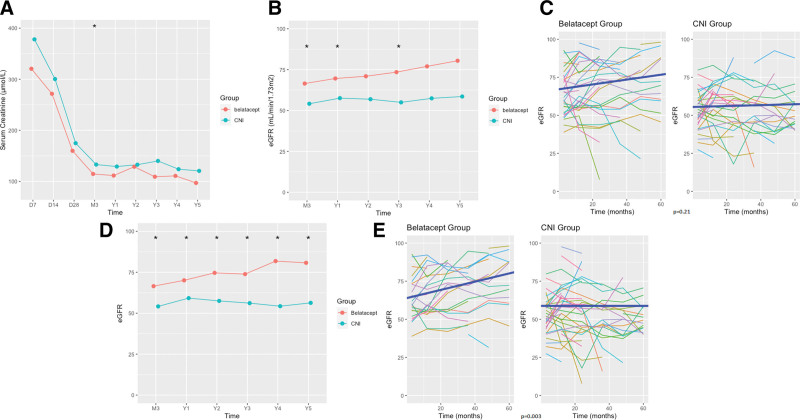
Comparison of kidney function (serum creatinine and eGFR) in kidney transplant recipients receiving belatacept or CNI. A, Comparison of serum creatinine in kidney transplant recipients in the belatacept group and CNI group. Serum creatinine is expressed in µmol/L. *P value <0.05 (Student’s *t*-test). B, Evolution of GFR in kidney transplant recipients in the belatacept group and CNI group. *P value <0.05. C, Individual evolution and slope of change of eGFR in kidney transplant recipients in the belatacept group and the CNI group. *P* value: comparison of slopes of change in eGFR using a mixed-linear model. D, Comparison of eGFR in patients receiving belatacept or CNI at the time of assessment (“on-treatment” analysis). *P value <0.05 (Student’s *t*-test). E, Individual evolution and slope of change of eGFR in patients receiving belatacept or CNI at the time of assessment (“on-treatment” analysis). *P* value: comparison of slopes of change in eGFR using a mixed-linear model. CNI, calcineurin inhibitors; D, day; eGFR, estimated GFR expressed in mL/min/1.73 m^2^; M, month; Y, year.

Kidney function (serum creatinine and eGFR from 3 mo posttransplantation) was followed during the first year and annually thereafter. All patients were followed up at least during the first posttransplantation year and until May 2022, but the number of patients that could be assessed decreased each year to reach 16 (CNI) and 15 (belatacept) at 5-y posttransplantation. We first compared renal function in the 2 groups at each time point: mean serum creatinine was significantly lower in the belatacept group only at 3 mo (114.6 ± 26.8 versus 132.9 ± 35.8 µmol/L, Student’s *t*-test, *P* = 0.032; Figure 1A; Table 4), and eGFR was significantly higher in the belatacept group at 3, 12, and 36 mo posttransplant but not thereafter (Figure 1B; Table 4). Second, we compared the slope of change in eGFR over time in the 2 groups using a mixed-effect model and was higher but not significantly in the belatacept group (+0.16 versus +0.03 mL/min/1.73 m² per month, *P* = 0.21; Figure 1C.

**TABLE 4.** Evolution of kidney function posttransplantation in belatacept and CNI groups

	Belatacept		CNI		*P* ^*a*^
	Mean (SD)	n	Mean (SD)	n	
eGFR M3	66.5 (16.80)	31	54.15 (15.44)	31	**0.007**
eGFR Y1	69.61 (19.34)	31	57.58 (18.20)	31	**0.020**
eGFR Y2	70.97 (24.90)	29	56.92 (18.78)	24	0.101
eGFR Y3	73.48 (23.54)	23	55.00 (20.39)	23	0.028
eGFR Y4	77.03 (28.62)	19	57.45 (19.83)	20	0.073
eGFR Y5	80.46 (21.15)	15	58.57 (23.29)	16	0.483

^*a*^Comparison of mean values was made by the test of student.

Bold indicates *P* values <0.05.

CNIs, calcineurin inhibitors; eGFR, estimated GFR by MDRD equation (expressed in mL/min/1.73 m^2^); M, month; n, number of available creatinine values per group; Y, year.

When we stratified on the type of donor, we observed a higher mean eGFR and a higher slope in eGFR change in the belatacept group but only for transplants from cDCD, no difference between the 2 treatment groups was observed for uDCD transplants (**Figure S2a and S2b, Table S1a and S1b, SDC**, http://links.lww.com/TXD/A638).

Given the number of patients who switched from one treatment to another, we compared kidney function in an “on-treatment” analysis, according to the treatment (belatacept or CNI) that patients were receiving at the timepoint of renal function analysis. We noted a significant and increasing difference in eGFR in favor of the belatacept group at each of these timepoints. At 5 y posttransplantation, this difference in eGFR reached 24.5 mL/min/1.73 m^2^ (80.7 ± 18.5 versus 56.3 ± 22.0 mL/min/1.73 m^2^, Table 5; Figure 1D). Moreover, the slope in eGFR change was significantly higher in the belatacept group (+0.27 versus 0.00 mL/min/1.73 m² per month, *P* = 0.003, Figure 1E).

**TABLE 5.** Comparison of kidney function in patients receiving belatacept or CNI at the time of assessment (“on-treatment” analysis)

	Belatacept group		CNI group		*P * ^*a*^
	Mean (SD)	n	Mean (SD)	n	
eGFR M3	66.5 (16.8)	31	54.2 (15.4)	31	**0.004**
eGFR Y1	70.1 (17.6)	25	59.2 (19.9)	37	**0.027**
eGFR Y2	74.6 (20.7)	22	57.5 (22.6)	31	**0.006**
eGFR Y3	73.9 (22.4)	21	56.1 (22)	25	**0.010**
eGFR Y4	81.8 (26.1)	18	54.3 (18.7)	21	**<0.001**
eGFR Y5	80.7 (18.5)	14	56.3 (22)	16	**0.003**

^*a*^Comparison of mean values was made by the test of student.

Bold indicates *P* values <0.05.

CNIs, calcineurin inhibitors; eGFR, estimated GFR by MDRD equation (expressed in mL/min/1.73 m^2^); M, month; n, number of available creatinine values per group; Y, year.

### Rejection Episodes

After a mean follow-up of 4.1 y, we noted 24 episodes of rejection in 15 patients in the belatacept group, and 6 episodes of rejection in 3 patients in the CNI group (Table 6). Importantly, 7 of these rejection episodes occurred after switch from belatacept to another immunosuppressive treatment (**Figure S1, SDC,** http://links.lww.com/TXD/A638).

**TABLE 6.** Incidence of rejections in the belatacept and CNI groups

	Episodes in the belatacept group	Episodes in the CNI group
Type of rejection		
TCMR—BL	11	0
TCMR—grade I	3	2
TCMR—grade II	2	0
MAR	3	0
Active AMR	3	3
Chronic active TCMR	1	0
Chronic active AMR	1	1
Total episodes of rejections	24	6
Including rejection on screening biopsies	6	0
Including rejection after immunosuppression switching	7	0

AMR, antibody-mediated rejection; BL, borderline; CNIs, calcineurin inhibitors; MAR, mixed acute rejection; TCMR, T cell–mediated rejection.

During the first-year posttransplantation, all biopsy-proven acute rejection episodes (n = 15) were observed in the belatacept group (2 episodes occurring after a switch). Importantly, more screening biopsies were performed during the first-year posttransplantation in the belatacept group (37 versus 24 in the CNI group) but only 6 rejection episodes were diagnosed on a screening biopsy in the belatacept group. These early episodes consisted of 11 acute cellular rejections (including 8 borderline changes), 2 mixed rejections and 2 AMRs. After the first year, 9 rejections were diagnosed in the belatacept group and 6 in the CNI group.

Overall, the difference in the type of rejection concerned the T cell–mediated rejection that were more frequently observed in the belatacept group, (21 episodes including borderline, mixed, grade I and II, versus 2 episodes in the CNI group), whereas 3 active AMR and 1 chronic active AMR were observed in each group.

### De Novo DSA

At a mean follow-up of 4.1 y, 8 patients developed 9 de novo DSA in the belatacept group and 7 developed 29 de novo DSA in the CNI group. DSAs were predominantly directed against class II and particularly the DQ locus in the 2 groups (Table 7). The mean MFI of the immunodominant DSA was lower in the belatacept group: 4375 ± 3455 versus 13 435 ± 8624 in the CNI group. One of the 8 patients in the belatacept group developed de novo DSA with MFI ≥10 000, versus 3 of the 7 patients in the CNI group. In the belatacept group, most de novo DSA (5/8) occurred after a switch to everolimus (1 patient) and tacrolimus (4 patients), for infectious or oncologic causes, after a mean delay of 7 mo (**Figure S1, SDC**, http://links.lww.com/TXD/A638).

**TABLE 7.** Characteristics of patients who developed de novo DSA in the belatacept and CNI groups

	Belatacept group	CNI group
	De novo DSA-positive patients	De novo DSA-positive patients
N (%)	8 (25.8)	7 (22.6)
Mean age, y	53.5	49.8
Male, n (%)	6 (75)	7 (100)
cPRA, n (%)		
<5%	5 (62.5)	3 (42.8)
5%–80%	3 (37.5)	4 (57.1)
>80%	0	0
De novo DSA detected before switch, n (%)	3 (37.5)	7 (100)
Graft failure, n (%)	1 (12.5)	3 (42.8)
Type of donor, n (%)		
cDCD	4 (50)	6 (85.7)
uDCD	4 (50)	1 (14.3)
Specificity, n (%)		
Class I	0	1 (14.3)
C	0	1
Class II	8 (100)	3 (42.8)
DR	1	0
DP	2	1
DQ	4	1
DR and DQ	1	1
Class I and II	**0**	3 (42.8)
B, DR, and DQ	0	1
A, B, C, DR, and DQ	0	1
A, B, DR, DQ, and DP	0	1
MFI max		
<3000	3	4
3000–10 000	4	0
>10 000	1	3

Data in the table are number of patients except for the age.

cDCD, donors after controlled circulatory death; CNIs, calcineurin inhibitors; cPRA, calculated panel-reactive antibody; DSA, donor-specific antibody; MFI, mean fluorescence intensity; uDCD, donors after uncontrolled circulatory death.

Two patients in the belatacept group (25%) developed AMR after de novo DSA occurrence, with no graft loss, whereas 3 (42.8%) patients in the CNI group experienced AMR, leading to graft loss in the 3 cases.

When we analyzed patients who remained on belatacept and CNI (“on-treatment” analysis), we observed that 3 patients developed de novo DSA in the belatacept group and 11 patients developed de novo DSA in the CNI group. We observed 2 episodes of active AMR after de novo DSA occurrence in the belatacept group and 4 episodes of active AMR after de novo DSA occurrence in the CNI group. No episodes of chronic AMR were observed in the belatacept group, whereas 2 episodes of chronic AMR occurred in the CNI group.

### Viral Infections

During the first-year posttransplantation, a positive BK viremia was observed in 22.6% of patients in the belatacept group versus 16.1% in the CNI group (*P* = 0.72). Three BK-virus nephropathies were diagnosed in the belatacept group, none in the CNI group.

CMV DNAemia was noted in 32.3% of patients in the belatacept group compared with 29.0% in the CNI group (*P* = 1) during the first year. Three CMV diseases were observed in each group.

In an “on-treatment” analysis, 2 BK-virus nephropathies were diagnosed in the belatacept group and 1 BK-virus nephropathy in the CNI group. One CMV disease was observed in the belatacept group versus 5 CMV diseases in the CNI group.

### Patient and Graft Survivals

**FIGURE 2.**
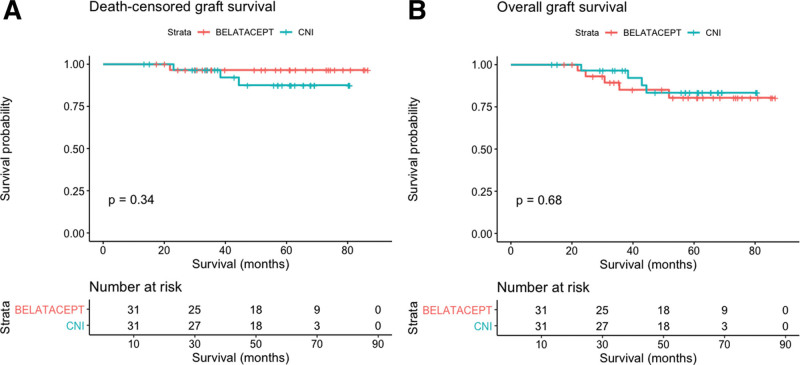
A, Death-censored graft survival in the belatacept and CNI groups. B, Overall graft survival in the belatacept and CNI groups. CNIs, calcineurin inhibitors.

Patient, death-censored and overall graft survivals were statistically similar in the 2 groups as a whole and stratified on the type of DCD (Figure 2A and B; **Figure S3, SDC**, http://links.lww.com/TXD/A638). There was no graft loss or death in the 2 groups during the first year.

In the belatacept group, there was 1 graft loss because of BKV nephropathy and 4 deaths caused by ischemic stroke, suicide, cardiorespiratory arrest and COVID pneumonia.

In the CNI group, 3 graft losses because of chronic AMR and 1 death from COVID pneumonia were observed.

## DISCUSSION

In our study including 62 paired-kidneys transplants from DCD, we aimed to compare the evolution of renal function with a belatacept- and CNI-based immunosuppression. We found no significant difference in DGF frequency and graft survivals between the 2 groups, but we observed a better renal function for the patients who remained on belatacept during the 5 y of follow-up.

Belatacept achieved progress as an alternative to CNI by improving graft function and preventing cardiovascular and metabolic side effects. This benefit was observed with transplants from standard criteria donors as well as from extended criteria donor.^5,7^ Belatacept has also been used more and more after conversion from CNI for nonalloimmune graft dysfunctions.^8^

The choice of belatacept in our center came from the results of the first studies in transplantation from DCD that showed increased incidence of primary nonfunction and DGF.^9,10^

We observed that the difference in DGF and the day of renal graft recovery posttransplantation was not significant, whatever the group of treatment or the donor’s type (cDCD or uDCD). In an intent-to-treat analysis, eGFR was higher in the belatacept group at 3, 12, and 36 mo but not thereafter. When we compared slopes of eGFR evolution in a mixed model, we observed no significant difference. However, after stratification on the type of donor, mean eGFR and slope of eGFR over time were significantly higher in the belatacept group but only for transplants from cDCD, not for uDCD. When we focused on patients who were on belatacept and CNI (on-treatment analysis), mean eGFR and slope of eGFR were significantly higher in the belatacept group. Interestingly, the mean slopes were positive in the belatacept group and close to 0 in the CNI group.

In our study, the majority of patients received tacrolimus (29/31), whereas in the initial studies, belatacept was compared with cyclosporine, still widely used.^4,5^ In a large registry study comparing belatacept to tacrolimus, renal function at 1 y was significantly higher among patients treated with belatacept, in the subgroup of BENEFIT-EXT eligible recipients.^11^ In the study by Cohen et al,^12^ patients treated with belatacept displayed a higher GFR at 1 y posttransplantation compared with tacrolimus.

As in many studies comparing belatacept to CNI,^4,11^ we observed higher rates of rejection with belatacept (24 episodes versus 6 episodes in the CNI group). These rejections were mainly early T cell–mediated rejections, despite thymoglobulin induction. The incidence of de novo DSA was similar in the 2 groups, but most of the DSA in the belatacept group developed after belatacept discontinuation and graft losses from chronic AMR were only observed in the CNI group. This is in accordance with the lower incidence observed with belatacept in BENEFIT studies.^13^

Regarding the rates of infections due to CMV and BK-virus, some studies found no difference in belatacept-treated patients compared with CNI groups,^7,14^ although other reported increased risk of CMV primary infection with belatacept.^15,16^ In our study, we found no difference regarding the incidence of CMV replication and BK viremia during the first year. We reported 3 BK-virus nephropathies in the belatacept group and a similar number of CMV diseases in the 2 groups.

After a mean follow-up of 50 mo, despite the higher incidence of T cell–mediated rejection with belatacept, we observed a similar overall and death-censored graft survival in the 2 groups. The better renal function on belatacept treatment did not translate into a benefit in terms of graft survival at this time of follow-up. In the long-term results of the BENEFIT study, despite an early gain of renal function with belatacept, the improvement in graft survival started to be observed after 60 mo.^7^

To our knowledge, our study is the first to assess specifically the safety and efficacy of belatacept compared with CNI (mostly tacrolimus) in kidney transplantation from uDCD and cDCD. In the BENEFIT-EXT initial study report, there were only 10% of kidney transplant from DCD, not studied separately.^6^ In a post hoc analysis of the BENEFIT-EXT, performed at 7 y after transplantation, a separated survival curve in the 55 DCD recipients showed a better overall graft survival in the belatacept “less-intensive” group compared with the cyclosporine group, but not in the belatacept “more-intensive” group.^17^ The gain in renal function observed in the 3 groups was not statistically different.

Our paired kidney design minimizes confusion and selection biases by comparing 2 recipients who received kidney transplants from the same donor and mainly differed by immunosuppressive protocol. A follow-up of 4 y was available for most of the patients; hence, an estimation of renal function was possible at medium term. We were able to check for immunological events by screening biopsies and systematic detection of de novo DSA.

The limitations of our study include its retrospective design and the relatively small size of our population. Particularly, we had less available serum creatinine values after 5 y. Importantly, an immunosuppression switch was made in a significant number of patients in the belatacept group (38.7%). This may limit the conclusions that can be drawn regarding influence of treatments, even if 88.7% of patients remained on the same treatment through the first-year posttransplantation. Our study is a “real-life” analysis, which reflects the medical practice in a transplantation center. An initial treatment by belatacept is likely to be discontinued by the nephrologists after an acute rejection episode (even borderline rejections) or an infection (bacteremia). These switches are not always medically indicated: 2 patients were switched for personal decision and another patient for travel reason (**Figure S1, SDC**, http://links.lww.com/TXD/A638).

In conclusion, the use of belatacept for kidney transplants from either uDCD or cDCD resulted in a better medium-term renal function for patients remaining on belatacept despite similar rates of DGF and higher rates of cellular rejection. This better renal function, associated to a lower incidence of de novo DSA under belatacept, supports the long-term use of belatacept in recipients of DCD transplants. Longer follow-up and a larger study are necessary to confirm these preliminary results in terms of graft survival.

## Supplementary Material


