# Supplementary material for: Use of a Belatacept-based Immunosuppression for Kidney Transplantation From Donors After Circulatory Death: A Paired Kidney Analysis
Source: Transplant Direct. 2024 Apr 11;10(5):e1615. doi: 10.1097/TXD.0000000000001615 (PMC11013701; doi:10.1097/TXD.0000000000001615)
Supplement: Supplementary file 1 [file txd-10-e1615-s001.pdf]

Supplementary material:

Figure S1: Follow up of kidney transplant recipients switched from Belatacept to another immunosuppressive drug in the Belatacept group.

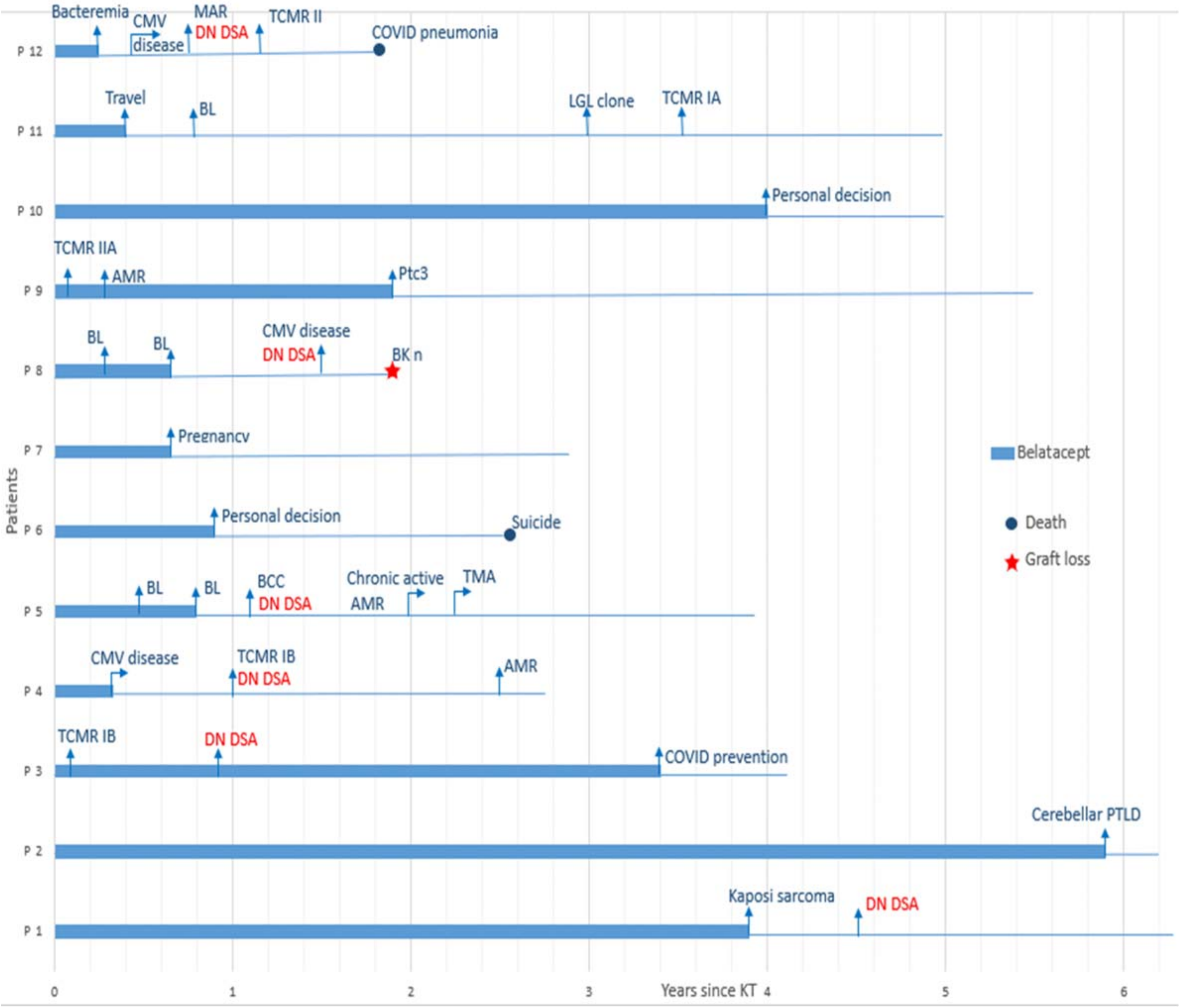

MAR: Mixed acute rejection; BL: Borderline rejection; DN DSA: De Novo Donor Specific Antibody; BCC: Basal cell carcinoma; PTLD: Post transplant lymphoproliferative disease; COVID: coronavirus disease; PTC3: Peritubular capillaritis; LGL clone: Large granular lymphocyte; BK n: BK virus nephropathy; TCMR: T cell mediated rejection; KT: Kidney transplantation

Figure S2a: Individual evolution and slope of change of eGFR in kidney transplant recipients from donors after controlled circulatory death in the Belatacept group and the CNI group (mixed effect model).

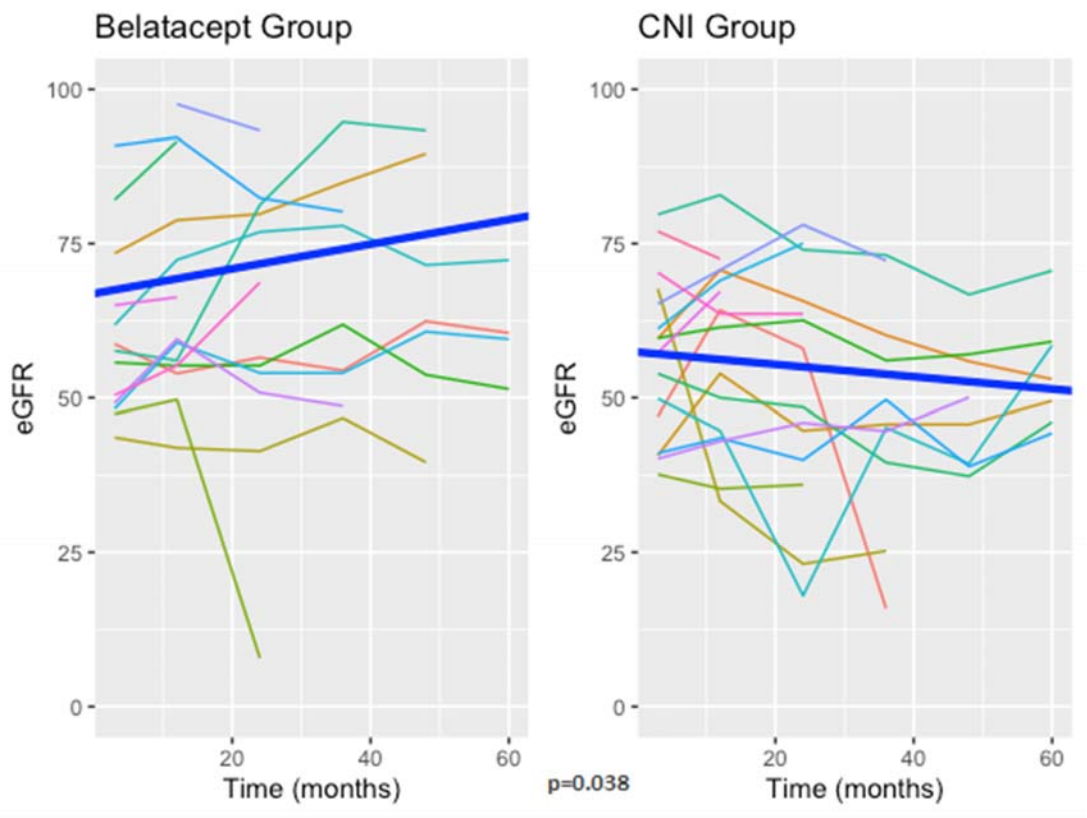

The slope was significantly higher in the belatacept group (+0.2 ml/min/1.73m² versus -0.1 ml/min/1.73m², p=0.038)

CNI: calcineurin inhibitors; D: day; M: month; Y: year

eGFR: estimated GFR expressed in ml/min/1.73 m^2, p value : comparison of slopes of change in eGFR using a mixed linear model

Figure S2b: Individual evolution and slope of change of eGFR in kidney transplant recipients from donors after uncontrolled circulatory death in the Belatacept group and the CNI group (mixed effect model).

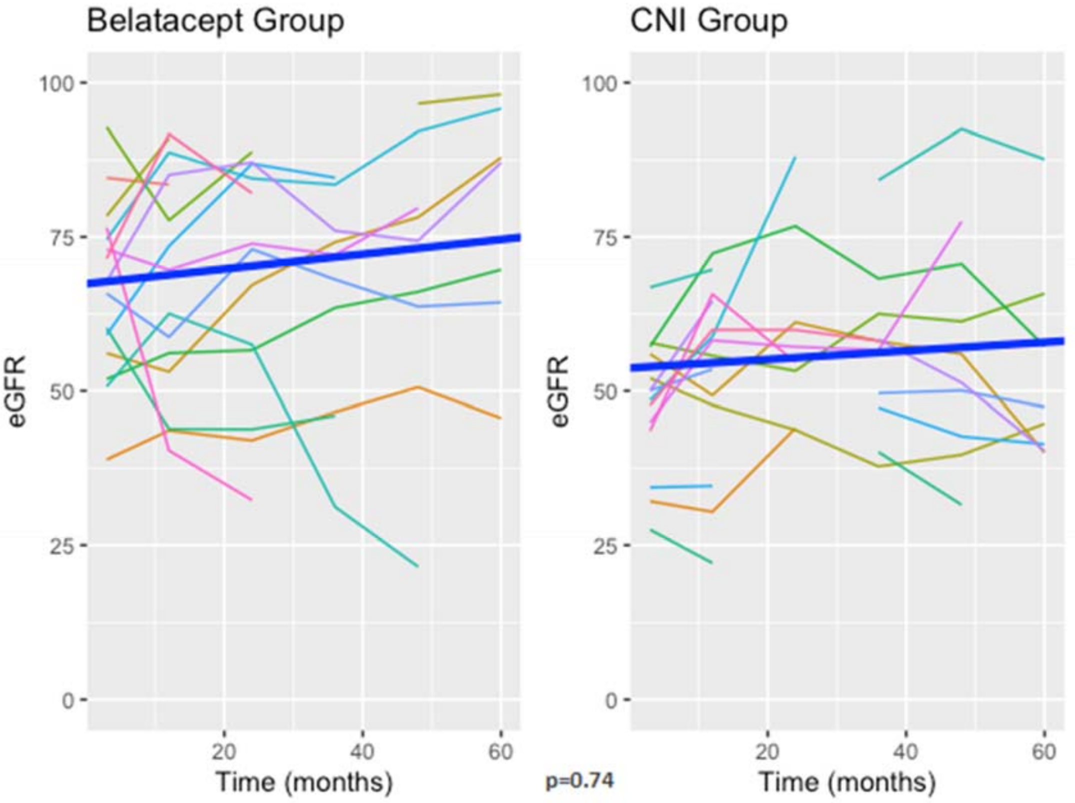

The slope was similar in the belatacept and in the CNI group (+0.12 ml/min/1.73m² versus +0.07 ml/min/1.73m², p=0.74)

CNI: calcineurin inhibitors; D: day; M: month; Y: year

eGFR: estimated GFR expressed in ml/min/1.73 m^2, p value : comparison of slopes of change in eGFR using a mixed linear model

Figure S3 a: Comparison of graft survival probability between kidney transplant recipients from donors after controlled death.

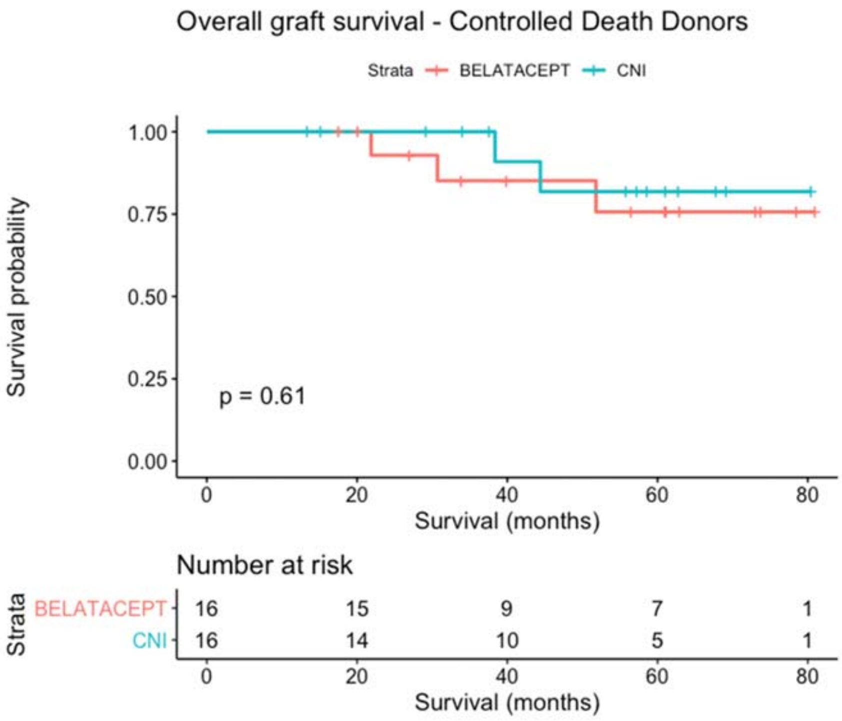

CNI: calcineurin inhibitors

Figure S3 b: Comparison of graft survival probability between kidney transplant recipients from donors after uncontrolled death.

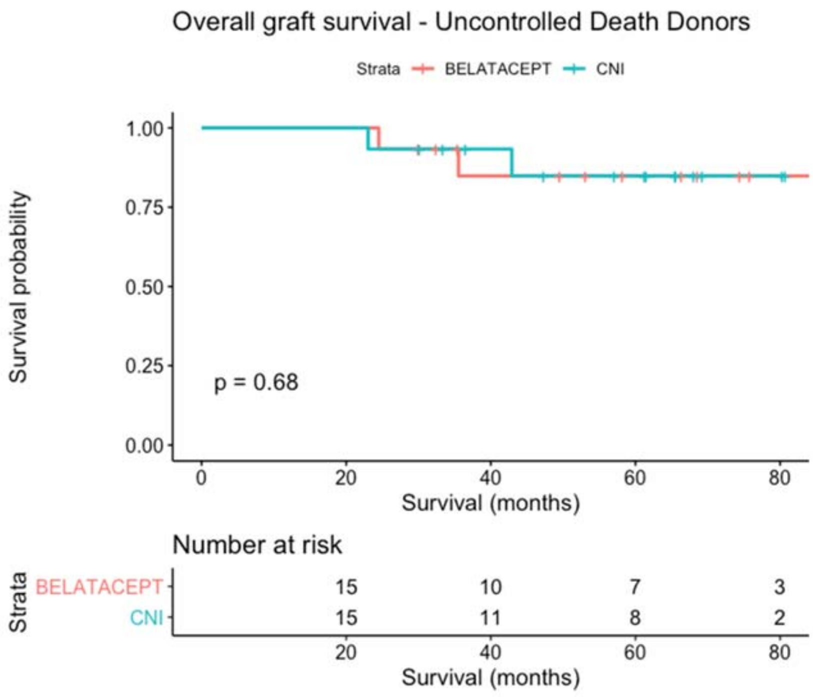

CNI: calcineurin inhibitors

Figure S3 c: Comparison of patient survival probability between kidney transplant recipients from Belatacept and CNI groups.

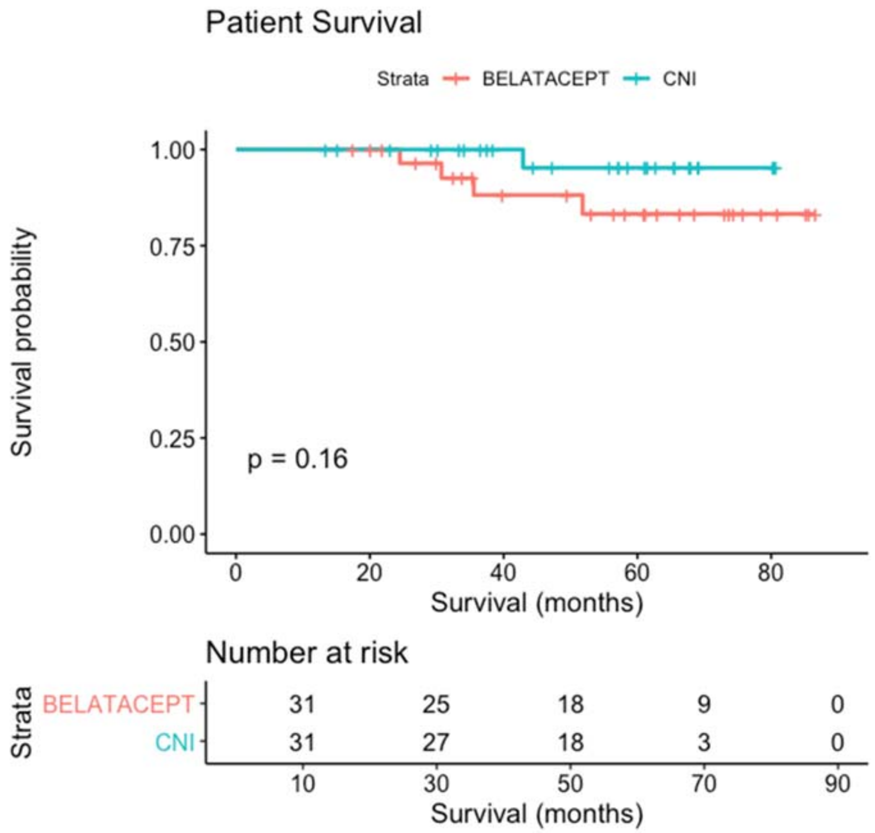

CNI: calcineurin inhibitors

Table S1 a: Evolution of kidney function post transplantation from donors after uncontrolled death in Belatacept and CNI groups.

uDCD	CNI		Belatacept		P value
	Mean (SD)	n	Mean (SD)	n	
S Cr M3	141.40 (41.23)	15	112.13 (25.09)	15	**0.015**
S Cr M12	133.80 (51.98)	15	112.47 (27.95)	15	0.097
S Cr Y2	113.50(29.54)	10	109.73 (36.28)	15	0.738
S Cr Y3	118.50 (32.79)	12	112.50 (42.19)	12	0.652
S Cr Y4	120.82 (41.01)	11	117.70 (68.46)	10	0.752
S Cr Y5	122.56 (38.02)	9	97.63 (24.76)	8	0.077
eGFR M3	51.39 (17.50)	15	66.79 (14.20)	15	**0.005**
eGFR M12	57.27 (21.84)	15	67.94 (18.13)	15	0.093
eGFR Y2	63.30 (17.49)	10	72.50 (22.42)	15	0.216
eGFR Y3	61.46 (21.35)	12	72.48 (24.78)	12	0.230
eGFR Y4	62.00 (23.55)	11	74.87 (27.96)	10	0.584
eGFR Y5	61.78 (30.47)	9	80.77 (19.01)	8	0.137

uDCD: donor after uncontrolled death; CNI: calcineurin inhibitors; D: day; M: month; Y: year; S Cr: Serum creatinine expressed in µmol/L; eGFR: estimated GFR expressed in ml/min/1.73 m^2 ; p value for Student’s t-test

Table S1 b: Evolution of kidney function post transplantation from donors after controlled death in Belatacept and CNI groups.

cDCD	CNI		Belatacept		p value
	Mean (SD)	n	Mean (SD)	n	
S Cr M3	125.00 (29.04)	16	116.88 (28.95)	16	0.399
S Cr M12	124.63 (35.89)	16	110.50 (29.93)	16	0.053
S Cr Y2	146.36 (59.99)	14	149.00 (159.96)	14	0.950
S Cr Y3	163.55 (93.20)	11	105.36 (29.63)	11	**0.029**
S Cr Y4	127.56 (18.68)	9	103.11 (39.07)	9	**0.049**
S Cr Y5	118.00 (18.70)	7	96.29 (26.02)	7	0.132
eGFR M3	56.75 (13.28)	16	66.23 (19.40)	16	0.129
eGFR M12	57.87 (14.74)	16	71.17 (20.88)	16	**0.028**
eGFR Y2	52.36 (18.93)	14	69.33 (28.09)	14	**0.042**
eGFR Y3	47.94 (17.55)	11	74.57 (23.27)	11	**<0.001**
eGFR Y4	51.89 (13.33)	9	79.42 (30.84)	9	**0.047**
eGFR Y5	54.44 (9.11)	7	80.11 (24.94)	7	0.060

cDCD: donor after controlled death; CNI: calcineurin inhibitors; D: day; M: month; Y: year; S Cr: Serum creatinine expressed in µmol/L; eGFR: estimated GFR expressed in ml/min/1.73 m^2 ; p value for Student's t-test
